# Teenager Substance Use on Reddit: Mixed Methods Computational Analysis of Frames and Emotions

**DOI:** 10.2196/59338

**Published:** 2025-02-19

**Authors:** Xinyu Zhang, Jianfeng Zhu, Deric R Kenne, Ruoming Jin

**Affiliations:** 1 College of Communication Michigan State University East Lansing, MI United States; 2 Department of Computer Science Kent State University Kent, OH United States; 3 College of Public Health Kent State University Kent, OH United States

**Keywords:** teenager, substance use, Reddit, emotional analysis, bidirectional encoder representations from transformers, BERT, frame approach

## Abstract

**Background:**

Adolescent substance use disorder is a pressing public health issue, with increasing prevalence as individuals age. Social media platforms like Reddit (Reddit Inc) serve as significant venues for teenagers to discuss and navigate substance use. Social media platforms, such as Reddit, serve as increasingly important spaces where teenagers discuss, share, and navigate their experiences with substance use, presenting unique opportunities and challenges for understanding and addressing this issue.

**Objective:**

This study aims to explore how teenagers frame substance-use discussions on the r/teenagers subreddit, focusing on their personal interpretations, causal attributions, and the social and psychological contexts that shape these online support groups. By identifying these interpretive frames, we aimed to better understand the complex drivers of adolescent substance use behavior and their potential interventions.

**Methods:**

Using natural language processing techniques, we analyzed 32,674 substance use–related posts from 2018 to 2022. A framing approach was used to identify and categorize prevalent themes, supplemented by emotional profiling using the EmoLLaMA-chat-13B model developed by Liu and colleagues.

**Results:**

In total, 7 primary frames emerged: normalization, risk awareness, social integration, autonomy and rebellion, coping mechanisms, media influence, and stigmatization. These frames varied in prevalence and were associated with distinct emotional profiles, highlighting the complex interplay between substance use and adolescent experiences. We observed that, for example, the normalization frame was often associated with a mix of sadness and anxiety, while the coping frame exhibited elevated levels of anger, sadness, and anxiety. These distinctive emotional landscapes associated with each frame reveal unique insights into the mental state of adolescents navigating substance use.

**Conclusions:**

The findings underscore the multifaceted nature of adolescent substance-use discussions on social media. Interventions must address underlying emotional and social factors as well as identity to effectively mitigate substance use disorder among adolescents. By understanding the frames teenagers use to interpret substance use, we can pave the way for more effective and personalized public health campaigns, and support services designed to resonate with adolescents’ unique lived experiences.

## Introduction

### Overview

Adolescent substance use disorder (SUD), characterized by a problematic pattern of substance use causing clinically significant impairment or distress, remains a significant public health concern [1]. Data from the monitoring of the future survey in the United States underscore a clear age-related increase in substance use during adolescence. Cannabis use and nicotine vaping remain particularly prevalent, with 29% of 12th graders, 17.8% of 10th graders, and 8.3% of 8th graders reporting cannabis use in the past year [2]. This trend reflects the progressive escalation of substance use as adolescents age, with nicotine vaping and cannabis being the most commonly used substances [2]. These findings align with the 2019 National Survey on Drug Use and Health, which reported a 4.5% prevalence of SUD among adolescents aged 12-17 years, rising to 14.1% among young adults aged 18-25 years. This further illustrates the role of age in substance use patterns [3]. A global meta-analysis found that older teenagers (aged 15-18 years) exhibit higher rates of substance use, with cannabis and methamphetamine becoming more prevalent as age increases [4]. This progression mirrors US patterns, where substance use typically escalates during mid-to-late adolescence.

The high prevalence of SUD among adolescents is particularly concerning owing to the potential risks and negative impacts associated with substance use [5-8]. SUD poses immediate health risks and can precipitate mental health challenges, infectious diseases like HIV or AIDS, and other negative societal impacts [9]. Families of those struggling with SUD often face profound disruptions, including strained relationships, communication breakdowns, and financial difficulties, all of which could further elevate the risk of SUD transmission across generations [5]. Despite these detrimental impacts, the stigma surrounding drug addiction frequently deters individuals from seeking help through traditional channels such as individual or group counseling [10]. In contrast, the anonymous nature of social media platforms like Reddit enables users to engage in discussions about sensitive topics such as substance-use experiences and seek support for recovery without fear of judgment. These platforms offer insights into a wide array of experiences, underscoring their importance in addressing the challenges of SUD [6].

An increasing number of studies have explored discussions of substance use and mental health on social media platforms such as Reddit. For example, research highlights Reddit’s role as a vital support network for opioid recovery, offering emotional and informational support [11,12]. Recent work using Reddit data reveals pandemic-related shifts in discussions about mental health, substance use, and lifestyle changes, highlighting correlations between the pandemic and shifts in health-related behaviors [13,14]. Other research focuses on specific emotional experiences related to substance use [15,16]. Collectively, this body of research illustrates the potential role of social media platforms in monitoring the evolving landscape of mental health and substance-use issues over time.

This research on discussions around substance use on Reddit has generally overlooked 2 critical areas. First, demographic factors, especially age differences, have largely been ignored. For instance, inhalant use is more prevalent among adolescents, whereas substances like nicotine, cannabis, alcohol, opioids, and benzodiazepines are more commonly used by individuals aged 21 to 30 years [17]. Existing studies of substance use discussions on Reddit have mainly focused on specific substances such as alcohol [12], marijuana [18], and opioids [19], disregarding the variations in substance use patterns and substance use among different age groups. Notably, there is a lack of focus on teenagers’ discussions about substance use, despite its significance as a growing concern.

Second, there is a substantial gap in understanding the interpretations of substance use by teenagers on these platforms. While existing studies have shed light on aspects such as language use [14], emotional expression [15], prevalent topics [14,20], and topical correlation [13], they fall short in investigating the deeper discourse within these conversations. In addition, research has highlighted how media portrays substance use primarily as an issue of criminal justice [21], but this perspective overlooks the importance of individual framing [22,23]. Particularly in matters with adverse personal and social outcomes, understanding teenagers’ personal interpretations and causal attributions is crucial for addressing the challenges associated with substance use.

To address these concerns, this study uses a framing approach to investigate teenagers’ discussions about substance use on Reddit, focusing on personal interpretations, causal attributions, and the broader social and psychological contexts influencing these conversations. Reddit is a social media platform consisting of numerous communities called subreddits, each dedicated to specific topics. The subreddit r/teenagers is a community where individuals aged 13 to 19 years share experiences and discuss issues relevant to their age group [24]. Goffman’s [25] concept of frames provides the theoretical foundation for our analysis. Frames are “schemata of interpretation” that enable individuals to “locate, perceive, identify, and label” occurrences within their life experiences and the world at large. By analyzing the frames used in r/teenagers discussions, we aim to reveal the interpretive lenses through which teenage users construct meaning around substance use. This approach allows us to uncover the narratives and attributions they use to make sense of the issue and how these framing processes relate to broader social and cultural contexts. Previous research has demonstrated the utility of framing theory in understanding health-related communications and behaviors. For example, studies have shown that framing influences individuals’ perceptions and responses to health messages [26]. Applying framing analysis to social media discussions provides a unique opportunity to explore how teenagers frame substance uses in their own words, offering insights that may not be accessible through traditional survey methods.

To systematically analyze the large volume of textual data available on Reddit, we use natural language processing (NLP) techniques. Automated NLP methods enable the processing of extensive datasets, capturing a more comprehensive picture of the discourse as compared with manual qualitative analyses. This approach enhances the reliability and generalizability of the findings by reducing potential biases introduced through selective sampling and human subjectivity [27]. The NLP techniques have been successfully used in previous studies to identify patterns and themes in social media data related to mental health and substance use [28], demonstrating their effectiveness in uncovering subtle linguistic and thematic patterns that might be overlooked in manual analysis. By leveraging these techniques, we aim to provide deeper insights into teenagers’ perspectives on substance use, which can inform targeted interventions and support strategies. Ultimately, we hope that our findings will contribute to the literature on adolescent mental health and substance-use prevention, supporting efforts to address these critical public health concerns.

### Literature Review

#### Teenagers’ Substance Use

Adolescent substance use is influenced by a complex interplay of peer dynamics, family environment, romantic relationships, and external factors. Peer influence, crucial in this context, manifests through peer selection and socialization, where adolescents align with peers having similar substance use behaviors and attitudes [8]. Concurrently, family dynamics significantly impact substance use, with factors like parenting styles, parental substance use, and family conflicts playing key roles [29,30]. Adolescents from authoritarian or conflicted family backgrounds, or those with parents who abuse substances, demonstrate a higher propensity for substance use [29].

In the context of romantic relationships, substance use can serve as a response to relationship-induced stress and may also contribute to adolescent dating violence [31]. For example, substance use often initiates at the onset of relationships and may persist or escalate as a coping mechanism for relationship stress and breakups. Research by Rothman et al [31] indicates that adolescents view alcohol consumption as intensifying aggression in dating violence by increasing irritability and anger. Moreover, external factors such as the COVID-19 pandemic have influenced substance use trends. While lockdown-induced reductions in social interactions have generally led to decreased substance use among teenagers [32], Alambo et al [13] observed an uptick in online support group discussions related to substance use disorders during the pandemic, indicating heightened awareness or concern.

Despite the critical insights these factors provide into adolescent substance use, research focusing on teenagers’ personal narratives within these contexts remains limited. A notable study in Australia identified four primary narratives among teenagers aged 13 to 15 years who were involved in alcohol and other drug programs: (1) viewing substance use as purposeful, (2) controlled, (3) a component of future competent use, and (4) not necessitating treatment [33]. However, a deeper understanding of how teenagers frame and make sense of substance use within their lived experiences and social contexts is still needed. This study aims to address this gap by using a framing approach [25] to analyze teenagers’ narratives on substance use. Frames represent interpretive lenses through which individuals make sense of and assign meaning to issues. By examining the frames used in teenagers’ discussions, we can gain insights into how they interpret and ascribe significance to substance use within their lived experiences and social environments.

#### Frame Approach

Frame research investigates the conceptual and interpretive mechanisms through which individuals comprehend the world. Rooted in interactionism and the School of Chicago, frame theory emphasizes the schemas that help individuals identify, perceive, and categorize experiences within their personal and broader environments [25]. These frames guide the interpretation of experiences and are shared by fellow actors, often taken for granted in daily interactions [25]. This study adopts an interpretive approach to frames, focusing on how individuals construct meaning and make sense of their lived experiences through cognitive schemas or “interpretive lenses.”

Understanding the collective framing processes used by teenagers is crucial for several reasons. First, in online support communities such as Reddit’s r/teenagers subreddit, users are not merely passive consumers but active creators of content. This user-generated content possesses the potential to influence and modify the interpretive frames of others, fostering a dynamic interplay between personal and collective framing processes [34]. As teenagers engage in discussions, share personal experiences, and collectively interpret substance use issues, collective action frames can organically emerge within the online support community.

Second, frames within social media discourse can significantly influence emotions, thereby shaping public understanding and engagement [35]. For instance, a study analyzing mental health frames on Twitter (X Corp) revealed 7 distinctive frames, including “Awareness,” “Feelings and Problematization,” “Classification,” “Accessibility and Funding,” “Stigma,” “Service,” and “Youth” [23]. These frames not only shape the conversation but also carry emotional weight that can vary in intensity, affecting the audience’s likelihood to engage civically [36]. The relationship between emotion and action extends to other areas of public discourse, such as substance use. For instance, a teenager’s Reddit post about recreational drug use can evoke a spectrum of emotions, ranging from concern to recognition of stress relief. Understanding these emotional responses is crucial for identifying discussions that may either hinder or promote positive societal change.

Several studies have individually examined the influence of specific frames surrounding substance use. One study explored how framing addiction as a brain disease within genetic and neuroscience research on nicotine addiction could undermine public health efforts, create unrealistic expectations regarding drug therapies, and divert attention from significant social factors [37]. Another study critiqued the framing of recreational substance use as potentially misleading and potentially detrimental [38]. While these studies highlighted issue-specific frames, they may not generalize across different contexts.

To date, there is a paucity of research investigating the frames and emotions associated with substance use discussions among teenagers. This gap is particularly concerning given the heightened vulnerability of this demographic to substance use disorder and its potential long-term repercussions. Exploring the prevalent frames and emotions in teenagers’ discourse surrounding substance use could yield valuable insights for developing tailored and effective prevention and intervention strategies. Consequently, the following research questions (RQs) are proposed: (1) RQ1: What frames do users in r/teenagers use to assign to substance use? (2) RQ2: Which of these frames on substance use are most prominent in these discussions? and (3) RQ3: Which emotions are commonly associated with individual frames in substance use discussions in r/teenagers?

## Methods

### Data Collection and Preparation

We analyzed originating posts from the r/teenagers subreddit, the largest online support forum for adolescents on Reddit, targeting users aged 13 to 19 years and boasting over 3 million subscribers. This subreddit offers a broad perspective on teenage life, making it a valuable resource for studying adolescent experiences, including substance use. Our analysis was confined to originating English-language posts, defined as the primary submissions made by users to the subreddit, rather than comments or replies to these submissions. This focus allowed us to prioritize the initial framing and self-expression by teenagers when discussing substance use.

To construct our dataset, we obtained 2,145,997 originating posts spanning from 2018 to 2022 through Reddit’s submission archives [39]. Posts related to substance use were identified through a systematically curated keyword-based methodology involving 3 distinct stages to ensure comprehensiveness and linguistic inclusivity. First, an initial list of keywords was compiled from authoritative sources, including the 2022 National Survey on Drug Use and Health by Substance Abuse and Mental Health Services Administration, which documents substances commonly used by adolescents; peer-reviewed studies such as Rutherford et al [40]; and the Drug-Use Insights web application [41], a resource providing substance-related terminology observed in social media contexts. Second, this list was expanded to include synonyms, abbreviations, and slang terms frequently used by adolescents, derived from an analysis of online support forums, social media platforms, and adolescent behavioral studies. This step was critical to capturing the diverse linguistic expressions used by teenagers when discussing substance use. Third, the expanded list underwent systematic refinement to eliminate terms deemed ambiguous, redundant, or irrelevant; for example, broad and nonspecific terms such as “party” were excluded, while slang terms directly related to substance use, such as “zaza” (referring to high-grade cannabis), were retained for their contextual relevance. This iterative process resulted in a final list of 150 terms, categorized into substance-use areas (refer to Table 1), enabling the identification of 32,674 substance-use-related posts within our dataset. Table 1 represents the finalized coding schema used to identify substance use-related posts from Reddit’s r/teenagers subreddit.

To prepare the extracted posts for analysis, we performed comprehensive text preprocessing to normalize the content and reduce noise. The steps included converting all text to lowercase; expanding contractions and replacing abbreviations using a custom dictionary tailored to adolescent slang (eg, “idk” to “I do not know”); removing HTML tags, numbers, punctuation, and special characters; normalizing elongated words and repeated characters (eg, “loooove” to “love”); removing stop words using natural language toolkit’s English stop word list supplemented with context-specific terms; and applying lemmatization to reduce words to their base forms. Duplicate posts were removed based on the cleaned text, and posts that were excessively short (fewer than 30 characters) after cleaning were excluded to ensure data quality.

**Table 1:** Substance-use keyword schema.

Categories	Keywords
Alcohol use	alcohol, beer, wine, liquor, whiskey, vodka, gin, rum, tequila, drunk, intoxicated, booze, hammered, tipsy, binge drinking, wasted, brandy, cocktail
Tobacco and nicotine use	nicotine, vape, vaping, tobacco, cigarettes, cigs, cigars, e-cigarettes, e-cigs, JUUL, dip, smoking, snuff, chewing tobacco, JUULing, hookah, nicotine patches, nicotine gum
Cannabis use	marijuana, weed, pot, THC^a^, cannabis, hash, hashish, ganja, skunk, dabbing, joints, blunts, reefer, Mary Jane, stoned, zaza (slang for high-grade cannabis), kush, 420, CBD^b^, hemp
Opioids (Prescription)	oxycodone, OxyContin, hydrocodone, Vicodin, Percocet, codeine, morphine, fentanyl, Dilaudid, Tramadol, painkillers, percs (slang for Percocet), Lorcet, Norco
Benzodiazepines	Xanax, Valium, Ativan, diazepam, clonazepam, Klonopin, alprazolam, lorazepam, temazepam, Restoril, midazolam, Dormicum, oxazepam, Serax, chlordiazepoxide, Librium, estazolam, Prosom, nitrazepam, Mogadon, benzos (slang for benzodiazepines), xans (slang for Xanax), flurazepam, Dalmane, triazolam, Halcion
Stimulants (Prescription)	Adderall, Ritalin, Vyvanse, Dexedrine, amphetamines, study drugs, addy (slang for Adderall)
Opioids (Illicit)	heroin, smack (slang for heroin), black tar, china white, fentanyl, chasing the dragon (slang for inhaling vaporized heroin or fentanyl)
Cocaine	cocaine, yayo (slang for cocaine)
Amphetamine-type stimulants	methamphetamine, meth, crystal meth
MDMA^c^	MDMA, ecstasy, Molly, candy flipping (slang for combining MDMA and LSD^d^)
Hallucinogens	LSD, shrooms, psilocybin, magic mushrooms, mescaline, DMT^e^, peyote, ayahuasca, 2C-B^f^, 2C-E^g^, 2C-I^h^, DOM^i^, STP^j^, DOB^k^, NBOMe^l^, 25I-NBOMe^m^, 25C-NBOMe^n^, 25B-NBOMe^o^, Iboga, Salvia, Psilocin, Bufotenin, 5-MeO-DMT^p^, Ergot, Ergine, Morning Glory, LSA^q^, Fly Agaric, Mescalito
Dissociative drugs	ketamine, Special K, PCP^r^, angel dust, DXM^s^, dextromethorphan, robo (slang for DXM abuse), nitrous oxide, whippets, methoxetamine, MXE^t^, Mexxy, eticyclidine, PCE^u^, DXO^v^, 2-oxo-PCE, O-PCE^w^, ibogaine, muscimol, tiletamine
Synthetic cannabinoids	K2^x^, fake weed, synthetic cannabinoids
Novel psychoactive substances	Flakka, research chemicals, RCs^y^, designer drugs

^a^THC: tetrahydrocannabinol.

^b^CBD: cannabidiol.

^c^MDMA: methylenedioxymethamphetamine.

^d^LSD: lysergic acid diethylamide.

^e^DMT: dimethyltryptamine.

^f^2C-B: 2,5-dimethoxy-4-bromophenethylamine.

^g^2C-E: 2,5-dimethoxy-4-ethylphenethylamine.

^h^2C-I: 2,5-dimethoxy-4-iodophenethylamine.

^i^DOM: 2,5-dimethoxy-4-methylamphetamine.

^j^STP: serenity, tranquility, and peace.

^k^DOB: 2,5-dimethoxy-4-bromoamphetamine.

^l^NBOMe: N-methoxybenzyl derivatives of the 2C family.

^m^25I-NBOMe: 2C-I-NBOMe (2-(4-Iodo-2,5-dimethoxyphenyl)-N-(2-methoxybenzyl)ethanamine).

^n^25C-NBOMe: 2C-C-NBOMe (2-(4-Chloro-2,5-dimethoxyphenyl)-N-(2-methoxybenzyl)ethanamine).

^o^25B-NBOMe: 2C-B-NBOMe (2-(4-Bromo-2,5-dimethoxyphenyl)-N-(2-methoxybenzyl)ethanamine).

^p^5-MeO-DMT: 5-methoxy-N,N-dimethyltryptamine.

^q^LSA: lysergic acid amide (D-lysergamide)

^r^PCP: phencyclidine

^s^DXM: dextromethorphan

^t^MXE: methoxetamine

^u^PCE: eticyclidine (N-ethyl-1-phenylcyclohexan-1-amine)

^v^DXO: dextrorphan

^w^O-PCE: N-ethyl-1-(2-fluorophenyl)-2-(methylamino)propan-1-one.

^x^K2: synthetic cannabinoids.

^y^RCs: research chemicals.

### Data Analysis

The labeling of Reddit posts was conducted through a systematic approach combining machine learning techniques with qualitative analysis. Initially, the BERT (Bidirectional Encoder Representations from Transformers)–based topic modeling algorithm identified topic clusters by capturing semantic relationships and patterns within the text. These clusters were provisionally labeled based on the most representative terms and phrases extracted by the algorithm.

To refine these labels, the author independently reviewed a representative sample of 30 posts from each cluster, conducting inductive coding to identify emergent themes, linguistic nuances, and contextual elements not fully captured by the algorithm. Following this independent analysis, collaborative discussions were held to reconcile differing interpretations, refine thematic categories, and develop operational definitions for each label. This iterative process ensured that the labels accurately represented the content and context of the posts, facilitating robust, contextually grounded labels tailored to the unique characteristics of the Reddit dataset.

Subsequently, the 34 identified topics were organized into 6 distinct frames, and the distribution of posts across these frames was calculated to assess their prevalence within the dataset. In the *Results* section, detailed descriptions and examples of these topics and frames are provided, illustrating their thematic relevance and enhancing the interpretability of the findings.

**Figure 1:**
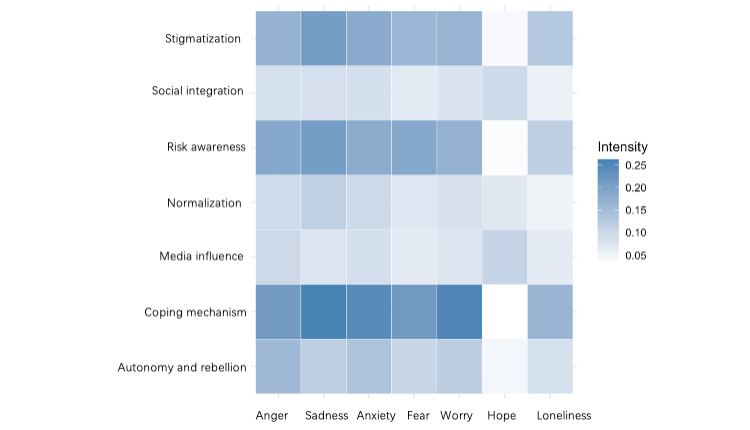
Frame-specific emotion intensity heat map.

To analyze the sentiment characteristics of posts, we used the EmoLLaMA-chat-13B model, a state-of-the-art tool within the EmoLLMs series built on Meta’s LLaMA2-chat-13B and fine-tuned using the affective analysis instruction dataset. This model notably outperforms previous state-of-the-art models on benchmarks like SemEval-2018, particularly in ordinal emotion intensity classification and emotion intensity regression [42]. In addition, it consistently outperforms advanced alternatives, including GPT-4 and ChatGPT, across various sentiment analysis tasks. It calculates emotion intensity scores for 7 core emotions: anger, sadness, anxiety, fear, worry, hope, and loneliness. These scores quantified the degree to which each emotion was expressed in the posts, allowing nuanced detection of emotional tones. For example, a post with scores of 0.33 for anger and 0.5 for sadness indicated moderate anger and substantial sadness, while other emotions remained minimal. This integrated approach facilitated a robust understanding of the emotional and thematic context of substance use discussions among adolescents. The average emotion scores for each frame were visualized using a heat map, as shown in Figure 1, further illustrating the emotional dynamics underlying these conversations. This heat map visualizes average intensity scores for 7 emotions across 7 substance-use frames identified in Reddit’s r/teenagers posts (2018-2022). Color intensity indicates the degree of emotion expression, ranging from 0.05 (lighter blue) to 0.25 (darker blue) based on EmoLLaMA-chat-13B model profiling.

### Ethical Considerations

The procedures conducted in this study adhered to ethical standards and were exempted from institutional review board review, as the research involved publicly available, anonymized data containing no identifiable information [28]. To further safeguard privacy, all posts included in the results were paraphrased to preserve their original meaning while ensuring that they could not be traced back to individual users.

## Results

### Overview

Table 2 displays the 3 topics identified by BERTopic. The “KeyBERT” column shows the top keywords associated with each topic, while the “Custom label” column was generated by our team through qualitative assessment. This process involved reviewing the top keywords and representative documents for each identified topic. Table 2 lists the 34 topics identified via BERTopic modeling from 32,674 Reddit posts on r/teenagers (2018-2022) related to substance use, along with their corresponding keywords (“KeyBERT”) and custom labels derived through qualitative analysis.

In accordance with various frames from other studies and the framing attribute of substance use, our team collaboratively analyzed and interpreted the themes to identify 7 overarching frames related to substance use within the r/teenagers subreddit discussions. These frames were normalization (17014/32674, 38.14%), risk awareness (1379/32,674, 4.22%), social integration (9573/32,674, 29.3%), autonomy and rebellion (1146/32,674, 3.51%), coping mechanism (5762/32,674, 17.64%), digital media influence (1310/32,674, 4.01%), stigmatization (1042/32,674, 3.19%). The identification of these frames highlights the interconnectedness of various factors influencing adolescent substance use, including social, familial, psychological, and cultural influences (refer to Figure 1).

**Table 2:** BERTopic-generated topics on adolescent substance use.

ID	KeyBERT^a^	Custom label	Percentage (N=32,674), n (%)
0	dad, mom, family	Family dynamics reinforcing substance use	6495 (19.88)
1	friend, girl, like	Peer relationships encouraging substance use	5093 (15.59)
2	personal, event, back	Stress coping through substance use	4099 (12.55)
3	school, teacher, class	Stress from school normalizing substance use	2552 (7.81)
4	juul, vape, vaping	Vaping as part of everyday life	1892 (5.79)
5	social, accept, drug	Substances as tools for peer acceptance	1689 (5.17)
6	birthday, party, friend	Party culture and group bonding	1359 (4.16)
7	feel, crisis, suicide	Mental health struggles and self-medication	1157 (3.54)
8	alcohol, taste, drinking	Teenage drinking as a social routine	1111 (3.40)
9	media, alcohol, rubbing	Social media promoting substance glamor	833 (2.55)
10	age, alcohol, drinking	Challenging legal drinking and vaping ages	753 (2.25)
11	anti-vaping, ads, campaign	Anti-vaping campaigns and social stigma	647 (1.98)
12	chat, drunk, social media	Drunken interactions on social media	618 (1.89)
13	concern, survey, test	Drug testing and its social implications	484 (1.48)
14	christmas, party, nicotine	Celebratory use of nicotine	412 (1.26)
15	iphone, phone, apple	Platforms encouraging substance trends	376 (1.15)
16	drunk, love, tomorrow	Romanticizing drinking in social settings	363 (1.11)
17	friend, social, group	Social situations facilitating substance use	350 (1.07)
18	died, death, driver	Death and accidents from substance use	284 (0.87)
19	covid, pandemic, virus	COVID-19 and awareness of substance risks	268 (0.82)
20	environment, health, pollution	Environmental impacts of substance use	261 (0.80)
21	juul, buy, access	Accessing restricted substances	209 (0.64)
22	sleep, drunk, alcohol	Using alcohol to manage sleep deprivation	196 (0.60)
23	religion, Muslim, god	Religion’s moral judgment of substance use	176 (0.54)
24	soda, nicotine, celebration	Alcohol as a comfort	176 (0.54)
25	poem, music, writing	Personal expression reflecting emotional struggles	134 (0.41)
26	drunk, friend, trend	Online portrayals of substance use	101 (0.31)
27	consent, rape, alcohol	Alcohol, consent, and youthful rebellion	101 (0.31)
28	russia, america, geopolitics	Sociopolitical resistance and substance use	101 (0.31)
29	game, play, video	Social connections through gaming	101 (0.31)
30	injury, surgery, shoulder	Health consequences of substance use	82 (0.25)
31	adderall, medication, price	The role of medication in adolescence	82 (0.25)
32	irish, culture, nationality	Cultural identity and substance perception	75 (0.23)
33	gay, trap, chromosome	LGBTQ+^b^ substance use and social stigma	62 (0.19)

^a^BERT: Bidirectional Encoder Representations from Transformers.

^b^LGBTQ+: lesbian, gay, bisexual, trans, and queer.

### Frame 1: Normalization

The normalization frame, accounting for 38.14% (12462/32674) of the discussions, reveals a substantial perception among adolescents that substance use is an accepted and routine part of daily life. This frame intertwines several topics: family dynamics reinforcing substance use (6495/32,674, 19.88%), stress from school normalizing substance use (2552/32,674, 7.81%), vaping as part of everyday life (1892/32,674, 5.79%), teenage drinking as a social routine (1111/32,674, 3.40%), and celebratory use of nicotine (412/32,674, 1.26%). Collectively, these topics illustrate how various facets of adolescents’ environments contribute to the normalization of substance use. Family behaviors and attitudes play a significant role, with substance use often perceived as a familial norm. Academic pressures lead to shared coping mechanisms like vaping and drinking, embedding these behaviors into daily routines. Social interactions further reinforce this normalization, as substance use becomes an expected component of communal activities and celebrations.

These shared understandings coalesce into collective action frames by fostering a collective identity where substance use is not only normalized but also encouraged. Adolescents internalize these behaviors as standard, leading to widespread participation and reinforcement within their social groups. One adolescent reflected, “In my family, everyone unwinds with a drink; so when I started drinking with friends, it just felt natural.” Another shared, “Vaping between classes is how we deal with stress—it’s just what we all do to get through the day.” Such narratives demonstrate how substance use is reinforced through family acceptance, peer practices, and shared coping strategies, solidifying its place in adolescent culture.

Emotional analysis within the normalization frame reveals moderate levels of sadness (0.092) and anxiety (0.112), indicating underlying emotional distress despite the outward acceptance of substance use. The topic “Stress from School Normalizing Substance Use” exhibits higher levels of sadness (0.14) and anxiety (0.18), suggesting that academic pressures significantly contribute to emotional turmoil and the adoption of substance use as a coping mechanism. Lower levels of positive emotions such as joy (0.072) imply that normalization does not necessarily enhance overall well-being. This suggests that while substance use is normalized, it may also be a response to deeper emotional challenges, further perpetuating its role in adolescents’ lives.

### Frame 2: Risk Awareness

The risk awareness frame accounts for 4.22% (1379/32,674) of the discussions, indicating a growing consciousness among adolescents about the negative consequences of substance use. This frame interconnects topics such as “Drug Testing and Its Social Implications” (484/32,674, 1.48%), “Death and Accidents from Substance Use” (284/32,674, 0.87%), “Environmental Impacts of Substance Use” (261/32,674, 0.80%), “COVID-19 and Awareness of Substance Risks” (268/32,674, 0.82%), and “Health Consequences of Substance Use” (82/32,674, 0.25%). Collectively, these discussions reflect an increased awareness of health risks, legal implications, and broader societal impacts associated with substance use.

The association among these topics illustrates how adolescents are developing a comprehensive understanding of the multifaceted risks involved. They express concerns not only about personal health but also about legal repercussions and the broader impact on their communities and the environment. This shared awareness leads to a collective reassessment of substance use, as adolescents weigh the perceived benefits against potential negative outcomes. One teenager remarked, “After my school started random drug testing, I realized how serious the consequences could be—not just for me but for my family too.” Another shared, “When I heard about someone from our town dying in a drunk driving accident, it hit me hard. It made me think about how my actions could affect others.”

These shared understandings coalesce into a collective action frame centered on caution and responsibility. Adolescents are not only reevaluating their own behaviors but are also contributing to a broader dialogue about the risks associated with substance use. The heightened emotional responses—elevated levels of sadness (0.188), anxiety (0.208), fear (0.188), and worry (0.181)—underscore the depth of their concern. As one individual noted, “With COVID-19 affecting people’s lungs, continuing to vape just doesn't seem worth it anymore.” Another stated, “I’m starting to see how smoking and littering impact the environment—we have to think about the bigger picture.” These narratives demonstrate a collective shift towards greater risk awareness and a desire to make more informed, responsible choices.

### Frame 3: Social Integration

Representing 29.30% (9573/32,674) of the discussions, the social integration frame underscores how adolescents use substance use to navigate social dynamics and achieve peer acceptance. This frame interweaves topics such as “Peer Relationships Encouraging Substance Use” (5093/32,674, 15.59%), “Substances as Tools for Peer Acceptance” (1689/32,674, 5.17%), “Party Culture and Group Bonding” (1359/32,674, 4.16%), “Social Situations Facilitating Substance Use” (618/32,674, 1.07%), “Drunken Interactions on Social Media” (363/32,674, 1.89%), “Romanticizing Drinking in Social Settings” (350/32,674, 1.11%), and “Social Connections Through Gaming” (101/32,674, 0.31%). Collectively, these topics illustrate the role of substance use as a means for group bonding and social interaction, revealing the complexities of adolescent social behavior.

The interconnections among these topics highlight a collective understanding among teenagers that substance use is integral to social belonging and acceptance. Adolescents often feel pressured to engage in substance use to align with peer norms and avoid isolation. One teenager shared, “If you’re not drinking at the party, it’s like you’re not really there. Joining in just makes you part of the group.” This sentiment reflects the drive to conform and the importance of participating in shared behaviors to maintain social bonds. Another adolescent noted, “Vaping with my friends between classes is just what we do; it’s our way of hanging out and decompressing together.” These narratives demonstrate how substance use becomes embedded in social rituals, reinforcing group cohesion and collective identity.

Moreover, the romanticization of drinking in social settings and the portrayal of “drunken interactions on social media” amplify this phenomenon, solidifying substance use’s role in adolescent culture. An individual observed, “Some of our best memories are made when we’re all a bit tipsy, laughing and just enjoying each other’s company.” Social media platforms often showcase these moments, further reinforcing the behavior. Despite this perceived enhancement of social connections, the emotional analysis reveals elevated levels of sadness (0.083), anxiety (0.085), and loneliness (0.054), suggesting that reliance on substances for social integration may not fully alleviate underlying emotional distress. This collective action frame reflects a complex interplay between the desire for social connection and the potential emotional costs associated with substance use, highlighting the need for a deeper understanding of adolescent social dynamics.

### Frame 4: Autonomy and Rebellion

Comprising 3.51% (1146/32,674) of the discussions, the autonomy and rebellion frame highlights how adolescents use substance consumption as a means of asserting independence and resisting authority. This frame intertwines topics such as “Challenging Legal Drinking and Vaping Ages” (735/32,674, 2.25%), “Accessing Restricted Substances” (209/32,674, 0.64%), “Sociopolitical Resistance and Substance Use” (101/32,674, 0.31%), and “Alcohol, Consent, and Youthful Rebellion” (101/32,674, 0.31%). Collectively, these topics reveal a shared desire among teenagers to assert control over their own decisions and to challenge societal restrictions, illustrating how substance use becomes a symbolic act of personal freedom and defiance.

The interconnections among these topics demonstrate a collective action frame where adolescents perceive substance use as a way to test boundaries and resist external control. One teenager asserted, “Why should the law tell me when I’m old enough to make my own choices? If I’m responsible enough to work and pay taxes, I should be able to decide if I want to drink.” This sentiment reflects a common challenge to age-based regulations, emphasizing a desire for self-determination. Another shared, “They keep making it harder to get vapes, but that just makes us more creative. It’s about proving that we won’t be controlled.” Such narratives underscore how obtaining and using restricted substances becomes a form of rebellion against perceived overreach by authorities.

Moreover, some adolescents connect their personal defiance to broader sociopolitical contexts. An individual remarked, “Using substances is our way of pushing back against a system that doesn’t listen to us. It’s not just about drinking or vaping; it’s about showing that we have a voice.” This perspective highlights how substance use can be intertwined with a larger critique of societal structures, reinforcing group identity through shared acts of resistance. Emotionally, this frame exhibits elevated levels of anger (0.156), sadness (0.114), and anxiety (0.136), reflecting the frustration and tension associated with challenging authority. Despite relatively low levels of hope (0.045) and loneliness (0.083), adolescents in this frame find solidarity in their collective pursuit of autonomy, united in their challenge to societal norms and expectations.

### Frame 5: Coping Mechanism

Accounting for 17.64% (5762/32,674) of the discussions, the coping mechanism frame illustrates how adolescents use substances to manage emotional stress and personal challenges. This frame integrates topics such as “Stress Coping Through Substance Use” (4099/32,674, 12.55%), “Mental Health Struggles and Self-Medication” (1157/32,674, 3.54%), “Using Alcohol to Manage Sleep Deprivation” (196/32,674, 0.60%), “Alcohol as a Comfort” (176/32,674, 0.54%), and “Personal Expression Reflecting Emotional Struggles” (134/32,674, 0.41%). Collectively, these topics reflect the significant role that emotional distress plays in adolescents’ decisions to engage in substance use, highlighting how substances become a means of coping with various stressors in their lives.

The interconnection among these topics underscores a shared understanding that substance use serves as a mechanism for self-medication and emotional regulation. Adolescents facing stress, mental health challenges, sleep deprivation, or emotional struggles turn to substances as temporary relief, seeking to alleviate negative emotions and escape from their problems. This collective behavior demonstrates how substance use becomes an ingrained coping strategy within this demographic, forming a collective action frame centered on managing personal hardships through substance use.

Teenagers’ narratives vividly capture this phenomenon, illustrating how their shared experiences coalesce into this collective frame. One adolescent admitted, “When school gets overwhelming, vaping is the only thing that helps me relax.” Another shared, “I can’t sleep at night, so sometimes I drink a little to help me drift off.” These statements reveal how substance use is perceived not merely as recreational but as a necessary tool for managing emotional and physical distress. Further emphasizing this point, an adolescent explained, “Dealing with anxiety is tough, and smoking weed is the only way I can feel normal.” Another added, “Writing songs while I’m high helps me express feelings I can’t put into words otherwise.” These authentic accounts highlight the reliance on substances to navigate their emotional landscapes and the collective interpretation of substance use as essential for coping.

Emotionally, this frame exhibits elevated levels of sadness (0.212), anxiety (0.262), and anger (0.244), indicating significant emotional distress among adolescents using substances to cope. Notably, the topic “Mental Health Struggles and Self-Medication” shows particularly high levels of sadness (0.23), anxiety (0.28), and loneliness (0.18), reflecting severe emotional turmoil. One post expressed, “My depression gets so bad that drinking is the only thing that numbs the pain, even if just for a little while.” Another shared, “I know relying on substances isn’t healthy, but it’s the only way I can get through the day.” The elevated levels of fear (0.215) and worry (0.253) further underscore the depth of their struggles. The relatively low level of hope (0.029) suggests a lack of optimism about their situations, reinforcing the reliance on substances for temporary relief. These shared sentiments illustrate how adolescents, through collective experiences and interpretations, normalize substance use as a coping mechanism for emotional distress. This collective action frame emphasizes the urgent need for supportive interventions, as teenagers mobilize around shared understandings to navigate their challenges.

### Frame 6: Media Influence

Accounting for 4.01% (1310/32,674) of the discussions, the media influence frame examines how social media and digital platforms shape adolescents’ perceptions of substance use through media socialization. This frame integrates topics such as “Social Media Promoting Substance Glamor” (833/32,674, 2.55%), “Platforms Encouraging Substance Trends” (376/32,674, 1.15%), and “Online Portrayals of Substance Use” (101/32,674, 0.31%). Collectively, these topics highlight the significant role of media in normalizing and glamorizing substance use among adolescents, thereby influencing their attitudes and behaviors.

The interconnections among these topics demonstrate the mechanisms by which media-driven norms operate. Adolescents are frequently exposed to portrayals of substance use as desirable and socially advantageous, fostering a perception of these behaviors as normative. This collective exposure leads to shared cognitive schemas and social comparisons that reinforce these impressions. The prevalence of substance-related content on social media platforms encourages adolescents to perceive substance use as an integral part of social identity and acceptance.

Teenagers’ narratives vividly capture this phenomenon, illustrating how their shared experiences coalesce into this collective frame. One adolescent remarked, “Scrolling through Instagram, it seems like everyone’s partying and having a great time with drinks in hand. It makes you feel like you’re missing out if you’re not doing the same.” Another shared, “All the popular kids are posting videos with vapes and flashy setups. It’s like you have to join in to be part of the trend.” These authentic accounts highlight the influence of media representations in shaping adolescents’ behaviors, as they adopt substance use to conform to perceived social expectations promoted by online support groups.

Emotionally, this frame is associated with elevated levels of sadness (0.096) and anxiety (0.086), suggesting that media portrayals contribute to negative effects among adolescents. The relatively high level of hope (0.108) indicates a complex emotional response, where optimism is intertwined with negative emotions. One user confessed, “I thought posting pics of me at parties would make me feel more connected, but I still feel alone when the likes stop coming in.” Another reflected, “Seeing everyone else having fun online support group makes me wonder if they’re really happy or just putting on a show like I am.” These shared sentiments underscore how media influence fosters a collective understanding that substance use is necessary for social inclusion, while also highlighting the emotional complexities involved. Through these shared experiences, adolescents collectively reinforce the media influence frame, which shapes their perceptions and actions regarding substance use.

### Frame 7: Stigmatization

The stigmatization frame, accounting for 3.19% (1042/32,674) of the discussions, centers on the moral and social judgments surrounding adolescent substance use, particularly within familial, religious, and cultural contexts. This frame includes topics such as “Anti-vaping Campaigns and Social Stigma” (647/32,674, 1.98%), “Religion’s Moral Judgment of Substance Use” (176/32,674, 0.54%), “LGBTQ+ Substance Use and Social Stigma” (62/32,674, 0.19%), “Cultural Identity and Substance Perception” (75/32,674, 0.23%), and “The Role of Medication in Adolescence” (82/32,674, 0.25%). These discussions collectively highlight how adolescents face criticism and moral condemnation from societal norms and authority figures, shaping their behavior and self-perception.

The interconnectedness of these topics underscores the pervasive experience of moral judgment, leading to the stigmatization that fosters guilt, shame, or defiance among adolescents. Antivaping campaigns often reinforce social ostracization, as reflected in the sentiment of one adolescent: “I feel like everyone looks down on me just because I vape—like I’m some kind of delinquent.” Such societal disapproval adversely impacts adolescents’ self-image, reinforcing isolation. Religious and cultural expectations further intensify this stigmatization, as illustrated by another adolescent’s experience: “In my community, drinking is considered a sin, but it’s hard when all my friends do it. I constantly feel torn between my beliefs and wanting to fit in.” These statements underscore the internal conflicts adolescents endure while negotiating personal desires and cultural values.

The stigmatization faced by lesbian, gay, bisexual, trans, and queer (LGBTQ+) adolescents is particularly acute, as they often experience dual layers of judgment concerning both their identity and substance use. One individual conveyed, “It’s hard enough being judged for who I am, but when they find out I smoke to cope, the judgment just doubles.” This compounded stigma exacerbates their sense of alienation. Similarly, the topic “The Role of Medication in Adolescence” reflects the stigmatization of prescribed medication use, even when these substances are essential for managing mental health. As one adolescent stated, “People think I’m weak because I take meds for my anxiety, but they don’t understand how much it helps.” These narratives demonstrate that stigmatization extends beyond illicit substances, affecting adolescents’ willingness to seek help and manage their health needs effectively.

The emotional dynamics of the stigmatization frame are marked by high levels of sadness (0.168), anxiety (0.183), and anger (0.161), indicating significant emotional distress due to societal and familial judgment. The topic “Religion’s Moral Judgment of Substance Use” exhibits particularly high levels of anger (0.256) and loneliness (0.145), suggesting that moral condemnation tied to religious beliefs exacerbates feelings of isolation and resentment. Moreover, the low levels of hope (0.039) within this frame indicate adolescents’ difficulty in finding optimism amid pervasive stigmatizations. One adolescent reflected, “Sometimes it feels like no matter what I do, I’ll never meet their expectations.” These narratives illustrate a collective experience of stigmatization that impedes well-being, reinforcing a shared perception of being marginalized and judged.

## Discussion

### Principal Findings

The findings of this study provide insights into interpretive frames adolescents use to construct and negotiate their perspectives on substance use, revealing intricate connections to broader sociocultural, emotional, and developmental contexts. At a conceptual level, the research underscores how adolescents engage with substances used as a mechanism to address sociocultural and identity-related challenges, situating these behaviors within collective-action frames that reflect both individual agencies and shared cultural dynamics. This framing highlights substance use not as an isolated behavior but as a socially embedded phenomenon with deep implications for adolescent development.

One key contribution is the identification of the normalization frame, which captures how substance use becomes embedded in adolescents’ everyday lives through familial, social, and institutional influences. Unlike previous studies that narrowly focused on peer influence or environmental factors, this research situates normalization within a wider cultural framework. It demonstrates how academic stress, parental modeling, and peer interactions collectively foster a shared perception that substances are used as routine. Such findings broaden the scope of existing literature by emphasizing the systemic reinforcement of these behaviors across multiple social domains.

The emergence of the risk awareness frame provides a compelling counter-narrative to normalization, illuminating adolescents’ critical reflections on substance-related risks despite their concurrent acceptance of such behaviors. This duality complicates simplistic dichotomies that frame adolescents as either deviant or compliant. Consistent with previous studies [43], this study shows heightened risk awareness, particularly in the wake of the pandemic, which intensified adolescents’ health consciousness and self-reflective capacities. The findings also resonate with Marinelli et al [44], who documented how the pandemic prompted adolescents to re-evaluate habitual behaviors, fostering a unique environment for behavioral reassessment.

The social integration frame further underscores the role of substance use in mediating adolescent social dynamics, positioning substances as tools for navigating group identity and peer acceptance. However, this narrative is complicated by findings that reveal underlying emotional vulnerabilities, such as sadness and anxiety; these findings suggest that while substance use may facilitate social connection, it also masks significant emotional distress. This creates a paradoxical feedback loop in which substance use both addresses and exacerbates emotional challenges, underscoring the need for interventions that address these deeper vulnerabilities.

Adding another layer to this analysis, the autonomy and rebellion frame portrays substance use as a symbolic act of resistance against societal and institutional constraints. This dimension situates adolescent substance use within a broader context of sociopolitical critique, where behaviors function as both a developmental milestone of individuation and a performative challenge to authority. These findings align with theoretical perspectives on youth subcultures, which emphasize resistance as a core characteristic while raising critical questions about the efficacy of punitive or prohibitive approaches to regulating adolescent behaviors.

The coping mechanism frame provides perhaps the most poignant insights, revealing that emotional distress (manifested in elevated levels of sadness, anxiety, and anger) is a pervasive driver of substance use among adolescents. This frame underscores the urgent need for mental health interventions that address the root causes of emotional distress rather than merely its behavioral manifestations. By situating these coping behaviors within a collective framework, the study highlights how adolescents share and normalize their struggles, entrenching substance use as a widely accepted strategy for emotional regulation. The lack of hope observed in this frame reflects systemic failures to provide adolescents with viable alternatives for managing psychological challenges, further reinforcing the importance of holistic and proactive support systems.

The media influence frame introduces a contemporary perspective, illustrating how social media amplifies cultural narratives around substance use by portraying it as glamorous or socially advantageous. While such portrayals exert considerable influence, the study also reveals adolescents’ growing skepticism and critique of these representations, reflecting an awareness of their constructed nature. This dual engagement suggests potential avenues for media literacy programs aimed at deconstructing harmful narratives and promoting more critical consumption of digital content.

Lastly, the stigmatization frame explores how moral and cultural judgments surrounding substance use profoundly affect adolescents’ experiences, contributing to heightened feelings of sadness, anxiety, and anger. This frame is particularly salient for marginalized groups, such as LGBTQ+ adolescents, who face compounded stigmatization based on both identity and behavior. The findings align with critiques of stigma-based deterrents, advocating instead for inclusive approaches that address systemic inequalities and provide supportive alternatives to punitive or judgmental responses. Therefore, the focus needs to be shifted away from blame and judgment and towards inclusive approaches that address inequality and offer alternative supportive environments, as has also been discussed in light of the pandemic by Marinelli et al [44].

Moreover, the findings of this study are particularly pertinent in light of the ever-evolving landscape of new psychoactive substances (NPSs), which pose a significant and novel challenge to public health and adolescent mental health in particular. While our research focuses on how teenagers frame and interpret substance use in online support discussions, it is critical to acknowledge the broader context in which this occurs. As Rinaldi et al [45] emphasize, the complexities of these NPSs (their diverse chemical structures and their ability to evade traditional drug enforcement) create novel risks. These substances are not just a theoretical concern; they can be found on illicit markets and, increasingly, online support groups, shaping the very discourse we analyzed on Reddit. Moreover, consistent with Meherali et al [46], the isolation and social disruption brought about by the COVID-19 pandemic, which potentially exacerbated online support interactions, have created new opportunities for illicit drug markets, as noted by Marinelli et al [44] and Negro et al [47]. This highlights the importance of online support group spaces, where adolescents are engaging in substance use discussions, as potential avenues for both risk and intervention. This understanding calls for a need to not only analyze the frames used to discuss substance use but also to address the underlying context of rapidly evolving substance markets and information streams that influence these narratives.

Theoretically, this study integrates these diverse frames into a cohesive framework, challenging reductionist models that view substance use as purely pathological or recreational. It highlights the interplay between individual agency and collective dynamics, offering a more comprehensive perspective on the social, emotional, and cultural dimensions of substance use. Practically, the findings advocate for targeted interventions tailored to the specific interpretive frames adolescents employ. For instance, strategies addressing normalization might focus on disrupting familial and peer modeling behaviors, while those targeting coping mechanisms should prioritize mental health resources and resilience-building initiatives.

By contextualizing substance use within broader sociocultural and emotional frameworks, this research deepens our theoretical understanding of adolescent behavior while offering actionable insights for fostering healthier environments. It calls for a shift in both policy and practice, emphasizing the importance of addressing systemic factors and respecting adolescents’ lived experiences to promote meaningful and sustainable change.

### Limitations and Futural Studies

While our study provides valuable insights into adolescent discourse on substance use, it is limited by the exclusive use of data from Reddit’s r/teenagers subreddit, an English-speaking online support forum. This introduces inherent biases, restricting the generalizability of our findings to the global adolescent population, as the perspectives captured predominantly reflect Reddit’s specific user demographic rather than a comprehensive view of teenage behaviors and attitudes worldwide. Moreover, our analysis does not fully contextualize substance use within the dynamics of the COVID-19 pandemic and the emergence of NPSs. Although we included keywords related to NPSs to capture emerging substances, we did not address the complex trafficking dynamics, pandemic-related changes, or broader mental health implications, which limits the depth of our findings concerning current societal influences on adolescent substance use.

Future research should address these limitations by incorporating more diverse data sources to enhance generalizability and by situating substance use within the context of the COVID-19 pandemic and NPS proliferation. Investigating discrepancies between online support discussions and offline attitudes can illuminate the influence of digital environments on real-world behaviors. In addition, examining the impact of significant societal events, such as public health crises and changes in drug legislation, on online support discourse could provide insights into the adaptability of adolescent attitudes and behaviors. By expanding the scope to include diverse data sources and contextual factors, future studies can deepen our understanding of adolescent substance use and inform more effective interventions. Such research would enhance the relevance and applicability of findings, ultimately contributing to solutions for the complex issues surrounding adolescent substance use.

### Conclusion

This study reveals that adolescents’ discussions about substance use on social media are predominantly framed around normalization, social integration, coping mechanisms, risk awareness, autonomy and rebellion, media influence, and stigmatization. The normalization and social integration frames indicate that substance use is perceived as a routine part of adolescent life, reinforced by family dynamics, peer relationships, and social media portrayals. Coping mechanisms highlight the use of substances to manage emotional distress, reflecting significant mental health challenges among youth. Although there is growing risk awareness, this consciousness does not consistently lead to behavioral change. The autonomy and rebellion frame illustrates substance use as a means for adolescents to assert independence and resist authority, while the stigmatization frame emphasizes the moral and social judgments contributing to feelings of isolation.

These findings underscore the multifaceted nature of adolescent substance use discussions and the necessity of addressing underlying emotional, social, and identity-related factors. Interventions should consider these frames to develop strategies that resonate with adolescents’ experiences and perceptions. By fostering supportive environments and promoting open dialogues about mental health and identity, health communication efforts can more effectively mitigate substance use among adolescents.

## Abbreviations

BERT: Bidirectional Encoder Representations from Transformers
LGBTQ+: lesbian, gay, bisexual, trans, and queer
NLP: natural language processing
NPS: new psychoactive substance
RQ: research question
SUD: substance uses disorder

## Data Availability

The datasets generated or analyzed during this study are available from the corresponding author on reasonable request.
